# Terrorism Catastrophizing and Sociodemographic Correlates Among Croatian Nursing Students: A Cross-Sectional Study

**DOI:** 10.3390/healthcare13182323

**Published:** 2025-09-16

**Authors:** Boris Ilić, Vesna Švab, Irena Kovačević, Biserka Sedić, Adriano Friganović, Ana Marija Švigir, Martina Smrekar, Štefanija Ozimec Vulinec, Samuel Justin Sinclair

**Affiliations:** 1 Faculty of Medicine, University of Ljubljana, 1000 Ljubljana, Slovenia; vesna.svab@mf.uni-lj.si; 2 Department of Nursing, University of Applied Health Sciences, 10000 Zagreb, Croatia; irena.kovacevic@zvu.hr (I.K.); biserka.sedic@zvu.hr (B.S.); adriano@hdmsarist.hr (A.F.); anamarija.svigir@zvu.hr (A.M.Š.); martina.smrekar@zvu.hr (M.S.); stefanija.ozimec-vulinec@zvu.hr (Š.O.V.); 3 National Institute of Public Health, 1000 Ljubljana, Slovenia; 4 Faculty of Health Studies, University of Rijeka, 51000 Rijeka, Croatia; 5 Brigham and Women’s Hospital, Boston, MA 02115, USA; drjustinsinclair@gmail.com; 6 Department of Psychiatry, Harvard Medical School, Boston, MA 02115, USA

**Keywords:** terrorism, catastrophizing, nursing students, Croatia, sociodemographic predictors, fear of terrorism, mental health

## Abstract

**Background/Objectives**: Fear of terrorism can impact psychological functioning and behavior even without direct exposure. Little is known about how anticipatory terrorism fears manifest among nursing students in European contexts. This study assessed terrorism catastrophizing among Croatian nursing students and examined sociodemographic predictors. **Methods**: A cross-sectional correlational study was conducted between October and December 2024 among 348 nursing students, using the validated Terrorism Catastrophizing Scale (TCS). Behavioral and habitual changes related to the terrorism threat were also measured. Non-parametric tests and bootstrapped regression analyses (1000 resamples) explored associations with sociodemographic variables. **Results**: Mean TCS score was 38.4 ± 8.0, indicating moderate catastrophizing, with subscale means of 16.8 (Helplessness), 11.7 (Rumination), and 9.8 (Magnification). Female students scored higher across all TCS measures (*p* < 0.001). Employment was associated with greater catastrophizing and behavioral changes, while urban residence was linked to fewer habitual and overall behavioral modifications. Higher income was associated with lower magnification. TCS scores correlated moderately with behavioral changes (rs = 0.27, *p* < 0.001). Non-parametric tests (Mann–Whitney U, Kruskal–Wallis, Spearman correlation) were applied due to non-normal distributions. **Conclusions**: Terrorism catastrophizing in this population is moderate and influenced by gender, employment, and residential context. Findings suggest targeted mental health support and tailored risk communication strategies may benefit nursing students in similar low-risk settings.

## 1. Introduction

Terrorism—broadly understood as the deliberate use of violence to create fear and drive political or ideological change—has emerged as a significant concern in the global security landscape, and its impact is not confined to nations that have suffered from direct attacks. In addition to populations residing in states directly affected by terrorist attacks, those in geographically distant or politically detached from such events can still experience the ripple effects through media coverage and international policy discourse [1,2]. While the physical consequences of terrorism are well documented, recent focus has shifted toward its psychological effects: anticipatory fear, anxiety, heightened perceptions of vulnerability, cognitive threat appraisal, and other psychological responses [3,4,5,6].

Among the constructs in this area, terrorism catastrophizing has gained particular attention. This refers to a cognitive pattern of thought characterized by persistent worry about future terrorist acts, often combined with a sense of helplessness in preventing or managing them [7,8]. Individuals with heightened fears about future terrorist acts may experience disproportionate anxiety, increased vigilance, and avoidance behaviors, even without direct exposure [5,8].

Much of the research on terrorism-related cognition and emotion has centered on populations in high-risk or directly impacted regions [9,10]. Far less is known about how these constructs appear in lower-risk, post-conflict European settings, including Croatia. This omission is important as Croatia’s population still carries the legacy of the 1990s war, a period of significant collective trauma [11]. Although domestic terrorism has not been a pressing issue in recent decades, it is plausible that residual historical trauma, combined with repeated media exposure to global terror incidents, could sensitize even young Croatian adults to perceive terrorism as a salient threat. In particular, younger generations who have grown up amid global war-on-terror narratives (despite living in a peaceful locale) may develop heightened threat appraisals or anxieties about terrorism [12]. However, empirical data on terrorism-related fear in such contexts are scarce.

Within this historical and social frame, nursing students stand out as a group worth studying. As future healthcare professionals, they are preparing for roles that involve responding to emergencies, as well as mass-casualty or terrorism-related scenarios. At the same time, they often face considerable psychological strain from academic pressure, anxiety, and burnout [13,14]. These stressors could potentially exacerbate worry tendencies or catastrophic thinking in the face of an added external threat such as terrorism.

This study addresses that gap by determining terrorism catastrophizing in Croatian nursing students using a validated translation of the Terrorism Catastrophizing Scale (TCS) [7]. It also examines whether the sociodemographic characteristics, such as gender, age, income, parental or relationship status, year of study, education level, and place of residence, are associated with variations in TCS scores. Prior research indicates that certain sociodemographic characteristics can influence how individuals perceive and respond to terrorism-related threats and stress. Gender, age, and parental or relationship status have been linked to variation in perceived vulnerability and distress [3,8]. Employment and income relate to the availability of psychosocial resources and vulnerability to stress [11,15]. In student populations, stress appraisal and coping capacities vary with academic stage, supporting the inclusion of year of study [13,16]. Additionally, residential context can also shape perceived safety and exposure to collective risk communication, with differences observed across urban/rural or socially vulnerable settings [17,18].

By focusing on this under-researched group in a low-risk yet historically traumatized context, the study contributes to the literature on terrorism-related mental health and offers insight for targeted interventions with future healthcare professionals.

## 2. Materials and Methods

### 2.1. Study Design

A cross-sectional correlational study was conducted to assess levels of terrorism catastrophizing and its associations with sociodemographic variables among nursing students in Croatia. A mixed-mode survey design was employed, combining in-person data collection in classroom settings with an online distribution strategy to enhance accessibility and reach. The study was prepared in accordance with the STROBE guidelines for cross-sectional studies (Supplementary Table S1).

### 2.2. Participants

The participants in this study were undergraduate and graduate nursing students enrolled in nursing programs across the Republic of Croatia. Recruitment involved both a classroom setting and online distribution through the snowball sampling technique, in order to increase the reach and allow participation from students across different universities and regions. Analyses were conducted on all available responses, and the number of valid responses is reported for each analysis.

Prior to conducting the study, the minimum required sample size was determined using a sample size calculator (α = 0.05, power = 0.80), which indicated n = 223 to detect a medium overall effect (f^2^ = 0.10) and n = 271 for a small–medium effect (f^2^ = 0.08). Our achieved sample (n = 348) exceeded these thresholds.

Participation in the study was voluntary, and all respondents provided informed consent prior to completing the questionnaire. Inclusion criteria were: (i) age ≥ 18 years, (ii) current enrollment in an undergraduate or graduate nursing program in Croatia, (iii) the ability to understand Croatian. Exclusion criteria were: (i) enrollment in a non-nursing program, (ii) inability to understand Croatian.

### 2.3. Data Collection

Data were collected between October and December 2024. A paper-based questionnaire was administered in classrooms, in addition to an online version of the identical questionnaire, created using the LimeSurvey platform v.6.5.4. A link to the online survey was distributed via internal mailing lists and social media platforms, with a request for participants to forward the link to their fellow nursing students in a snowball sampling manner.

### 2.4. Instruments

A two-part instrument was used in this study. The first part collected sociodemographic information, and the second part consisted of the Terrorism Catastrophizing Scale (TCS), used to assess cognitive responses related to perceived terrorism threats.

Participants provided the following sociodemographic data: age, gender, year of study, relationship status, parental status, household income, and place of residence (rural, small settlement, medium-sized, urban). These variables were selected based on previous research identifying potential sociodemographic correlates of psychological outcomes among university students and healthcare professionals.

The Terrorism Catastrophizing Scale (TCS), originally developed by Sinclair and LoCicero (2007) [7], is a 13-item self-report questionnaire developed to measure future-oriented catastrophic thinking specific to terrorism. The scale assesses the extent to which individuals ruminate about, anticipate, or feel helpless in response to the perceived threat of terrorism. Items are rated on a 5-point Likert scale (1 = strongly agree to 5 = strongly disagree), with total scores ranging from 13 to 65. TCS total scores were calculated as the sum of all 13 items, and subscale scores were calculated as the sum of their respective items, with higher scores indicating greater catastrophizing. Most items require reverse scoring (1, 2, 3, 4, 6, 7, 8, 9, 10, 12, 13) before analysis. The TCS comprises three cognitive subscales:

- Helplessness (5 items: 2, 4, 7, 11, 13)—captures a perceived inability to protect oneself or respond effectively in the event of a terrorist attack.
- Rumination (5 items: 1, 3, 5, 9, 12)—reflects persistent, intrusive, or exaggerated thoughts about terrorism and its consequences.
- Magnification (3 items: 6, 8, 10)—measures the tendency to exaggerate the severity of the impact of a potential terrorist attack.

In addition to the TCS, terrorism-related behavioral modifications were assessed using two subscales (Behavioral, 5 items; Habitual, 4 items) originally developed by Sinclair and LoCicero [7]. Items were rated on a 5-point scale (1 = no change, 5 = complete change), with higher scores indicating more extensive modifications. In our sample, internal consistencies for these subscales were acceptable to good (Behavioral α = 0.718, Habitual α = 0.786, combined behavioral change scale α = 0.810).

The questionnaire was translated and culturally adapted by a panel of nursing educators, in accordance with the cross-cultural adaptation framework developed by Beaton et al. [19]. An independent native English speaker back-translated the instrument, and the two versions were compared for semantic equivalence. A pilot test followed, involving 20 nursing students who identified areas of ambiguity or uncertainty. Based on this feedback, the final version of the questionnaire was produced and used in the study.

### 2.5. Data Analysis

Statistical analyses were conducted using IBM SPSS version 30.0.0. (IBM Corp., Armonk, NY, USA). Due to very small subgroup sizes, certain categories were merged for analysis (e.g., unemployed students, n = 3, were combined with the student group; widowed individuals, n = 2, were combined with divorced). Internal consistency of all scales and subscales was evaluated using Cronbach’s alpha.

The analytical approach proceeded systematically from descriptive to inferential statistics. Initial descriptive analyses examined means, standard deviations, medians, and interquartile ranges for all study variables. Normality testing was conducted using both Kolmogorov–Smirnov with Lilliefors correction and Shapiro–Wilk tests. Given significant deviations from normality across all variables, non-parametric analyses were employed for group comparisons, including Mann–Whitney U tests for binary variables and Kruskal–Wallis tests for categorical variables with multiple groups. Spearman’s rank-order correlations examined associations between continuous variables. Finally, bootstrapped multiple regression analyses with 1000 resamples and dummy coding were conducted to identify sociodemographic predictors of terrorism catastrophizing and behavioral change measures while accounting for non-normal distributions and providing robust confidence intervals.

The bootstrap analyses employed bias-corrected and accelerated (BCa) 95% confidence intervals, with reference categories established as follows: Gender = Male, Education = Secondary school, Residence = Rural area, Employment = Student/unemployed, Income ≤ €677, Children = No, and Marital status = Single.

### 2.6. Ethical Considerations

The study was approved by the Ethics Committee of the University of Applied Health Sciences in Zagreb, Croatia (Class: 602-04/17-18/585, No. 251-379-1-17-02), and conducted in accordance with the Declaration of Helsinki.

Participants were informed about the purpose of the study, and informed consent was obtained. Participation was voluntary and anonymous. Students were informed about the study’s purpose, and only those who provided informed consent (on the survey’s cover page or online form) could proceed. No incentives were offered for participation. Participants did not receive any incentives for participating in the research. Permission to use the TCS instrument was obtained from its original author prior to data collection.

## 3. Results

A total of 362 students initiated the survey. After excluding incomplete responses, the final sample comprising 348 students for the TCS measures; of these, 335 provided complete data for the behavioral change subscale and 334 for the habitual change subscale. Participants had a mean age of 24.0 years (SD = 6.0, range = 18–53 years, Mdn = 22, IQR = 21–24). The majority of participants were in their second and third years of undergraduate study, accounting for 31.0% (n = 108) and 37.6% (n = 131), respectively, while first-year undergraduate students represented 24.1% (n = 84) of the sample. Fourth and fifth-year graduate students comprised smaller proportions at 3.4% (n = 12) and 3.7% (n = 13), respectively. The sample demonstrated a pronounced gender imbalance, with females comprising 85.9% (n = 299) and males 14.1% (n = 49) of participants. This distribution is consistent with the demographic profile of nursing programs, where female students represent the vast majority of enrolments [20,21,22]. While this accurately reflects the underlying population, the small male subgroup may limit the robustness of gender comparisons.

The vast majority of participants (92.2%, n = 321) had completed secondary school as their highest educational level, while 7.8% (n = 27) had completed college or university education. Participants were predominantly from urban areas, with 43.7% (n = 152) residing in locations with populations exceeding 50,000 people. Rural residents comprised 21.0% (n = 73), while those from medium-sized settlements (5000–50,000 people) and small settlements (<5000 people) represented 20.1% (n = 70) and 15.2% (n = 53), respectively.

Employment status was relatively evenly distributed, with 54.0% (n = 188) classified as student/unemployed and 46.0% (n = 160) employed. Income distribution showed that 43.1% (n = 150) earned less than 677 euros monthly, while 36.2% (n = 126) earned between 1001 and 2000 euros. Only 6.9% (n = 24) reported monthly income exceeding 2000 euros. The majority of participants were childless (87.6%, n = 305) and single (58.9%, n = 205), with 21.0% (n = 73) cohabiting with a partner and 11.5% (n = 40) legally married (Table 1).

Analysis of individual TCS items revealed varying levels of terrorism-related catastrophizing thoughts among participants. The highest mean scores were observed for items addressing perceived helplessness, with “Little I can do to protect myself from terrorism” (M = 3.6, SD = 1.1, Mdn = 4.0) and “Feel powerful in keeping self-safe” (M = 3.5, SD = 1.0, Mdn = 3.0) receiving the strongest endorsement. Items related to future concerns also showed elevated scores, including “Worry terrorism will worsen with time” (M = 3.5, SD = 1.1, Mdn = 4.0) and “Feel helpless protecting myself” (M = 3.4, SD = 1.0, Mdn = 3.0).

Conversely, items measuring active preoccupation with terrorism demonstrated lower mean scores. “Frequently preoccupied with terrorism” received the lowest endorsement (M = 1.9, SD = 1.0, Mdn = 2.0), followed by “Often dwell on future terrorism” (M = 2.0, SD = 1.0, Mdn = 2.0). The item “Threat of terrorism does not enter my mind often” also showed relatively low scores (M = 2.4, SD = 1.1, Mdn = 2.0).

Examination of TCS subscales revealed that Helplessness demonstrated the highest mean score (M = 16.8, SD = 3.6, Mdn = 17.0), followed by Rumination (M = 11.7, SD = 4.0, Mdn = 12.0) and Magnification (M = 9.8, SD = 2.8, Mdn = 10.0). The total TCS score averaged 38.4 (SD = 8.0, Mdn = 38.0, IQR = 34.0–44.0), indicating moderate levels of terrorism catastrophizing across the sample. The distribution of scores appeared relatively normal, as suggested by the close proximity of mean and median values across most measures. Descriptive statistics for individual TCS items and subscales are presented in Supplementary Table S2.

Analysis of terrorism-related behavioral and habitual changes revealed generally low levels of modification across most domains, with participants reporting minimal alterations to their daily activities and lifestyle choices. Among behavioral changes, the most pronounced modification was observed in “Interacting with individuals of Middle Eastern or Arab descent” (M = 2.2, SD = 1.5, Mdn = 1.0), though this still represented relatively modest change on the 5-point scale. “Flying on commercial airplanes” showed the second-highest level of behavioral modification (M = 1.9, SD = 1.4, Mdn = 1.0), while other behavioral domains demonstrated minimal change, including “Using public transportation” (M = 1.5, SD = 1.0, Mdn = 1.0), “Going to public places” (M = 1.4, SD = 0.9, Mdn = 1.0), and “Voting in elections” (M = 1.4, SD = 1.0, Mdn = 1.0).

Regarding habitual changes, “News consumption habits” demonstrated the highest level of modification, followed by both “Vacationing preferences” and “Residential decisions”. Changes in “Working or attending school” showed the lowest modification. The Behavioral subscale yielded a mean score of 8.3 (SD = 4.0, Mdn = 7.0, IQR = 5.0–10.0) out of a possible 25, while the Habitual subscale averaged 7.5 (SD = 3.7, Mdn = 6.0, IQR = 4.0–10.0) out of 20. The Total Behavioral scale mean was 15.7 (SD = 6.7, Mdn = 14.0, IQR = 10.0–19.0) out of 45, indicating that participants generally maintained their pre-terrorism threat behaviors and habits with minimal modification. Notably, several behavioral items had a median of 1.0 with an IQR of 1.0–1.0 (e.g., ‘Using public transportation’, ‘Going to public places’, ‘Voting in elections’), indicating minimal absolute change for a large portion of the sample (Table 2).

Reliability analysis demonstrated adequate to good internal consistency across all measured scales. The Terrorism Catastrophizing Scale (Total) exhibited good reliability (α = 0.827), with its subscales showing acceptable to good consistency: Magnification (α = 0.820), Rumination (α = 0.777), and Helplessness (α = 0.715). The behavioral change measures also demonstrated satisfactory reliability, with the Total Behavioral scale achieving good internal consistency (α = 0.810). The Habitual subscale showed good reliability (α = 0.786), while the Behavioral subscale demonstrated acceptable consistency (α = 0.718). All Cronbach’s alpha values exceeded the conventional threshold of 0.70 for acceptable reliability. These reliability coefficients support the internal consistency of the study instruments and suggest that the scales reliably measured their respective constructs within this sample. (Table 3)

Normality testing revealed significant deviations from normal distribution across all study variables. Both the Kolmogorov–Smirnov test with Lilliefors correction and the Shapiro–Wilk test indicated non-normal distributions for all terrorism catastrophizing and behavioral change measures (all *p* < 0.05). The TCS Total scale showed the least deviation from normality among catastrophizing measures (Shapiro–Wilk W = 0.990, *p* = 0.015), while its subscales demonstrated more pronounced non-normality: Magnification (W = 0.958, *p* < 0.001), Helplessness (W = 0.982, *p* < 0.001), and Rumination (W = 0.974, *p* < 0.001). Behavioral change measures exhibited the most substantial departures from normality, with the Behavioral subscale showing the greatest deviation (W = 0.809, *p* < 0.001), followed by the Habitual subscale (W = 0.852, *p* < 0.001) and Total Behavioral scale (W = 0.872, *p* < 0.001). These findings necessitated the use of non-parametric statistical analyses for subsequent hypothesis testing. (Supplementary Table S3)

Analysis of group differences revealed several significant associations between sociodemographic characteristics and study variables. Gender emerged as a significant predictor of terrorism catastrophizing, with females reporting significantly higher TCS Total scores (M = 38.9, SD = 7.9) compared to males (M = 35.0, SD = 7.9, *p* = 0.001). However, gender differences were not significant for behavioral changes (*p* = 0.856).

The place of residence demonstrated significant associations with behavioral changes (*p* = 0.010) but not with terrorism catastrophizing (*p* = 0.075). Participants from small settlements (<5000 people) and medium-sized settlements (5000–50,000 people) reported the highest behavioral change scores (M = 17.4, SD = 8.0 and M = 17.1, SD = 6.9, respectively), while urban residents (>50,000 people) showed the lowest scores (M = 14.5, SD = 5.8).

Employment status significantly influenced behavioral changes (*p* < 0.001), with employed participants reporting higher behavioral modification scores compared to student/unemployed individuals. However, this category included diverse forms of employment, and information on job type or working hours was not collected. This heterogeneity limits interpretation and should be addressed in future research, particularly by distinguishing between healthcare and non-healthcare employment. Similarly, average monthly income showed significant associations with behavioral changes (*p* = 0.010), with middle-income earners (1001–2000 euros) demonstrating the highest behavioral change scores. No significant differences were observed for year of study, educational level, having children, or marital status for either terrorism catastrophizing or behavioral changes (all *p* > 0.05). (Table 4)

Correlation analysis revealed a significant positive relationship between terrorism catastrophizing and behavioral changes (rs = 0.27, *p* < 0.001), indicating that individuals with higher levels of terrorism-related catastrophic thinking were more likely to modify their behaviors and habits. Age showed weak, non-significant correlations with both TCS Total scores (rs = −0.06, *p* = 0.309) and Behavioral Scale scores (rs = 0.06, *p* = 0.275), suggesting that terrorism-related responses were relatively consistent across age groups within this sample. (Table 5)

Bootstrapped regression analysis for the Magnification subscale revealed that employment status and monthly income were the only significant predictors among the sociodemographic variables examined. Employed participants demonstrated significantly higher magnification scores compared to student/unemployed individuals (B = 1.235, 95% CI [−0.09, 2.41], *p* = 0.033). Regarding income, participants earning more than €2000 monthly showed significantly lower magnification scores relative to those earning less than €677 (B = −1.410, 95% CI [−2.76, 0.12], *p* = 0.049). No statistically significant difference was observed for the €1001–2000 income group (B = −1.227, 95% CI [−2.48, 0.10], *p* = 0.061), though the direction of the effect suggested a potential negative relationship between higher income levels and terrorism magnification. Gender showed a non-significant trend (B = 0.744, 95% CI [−0.09, 1.71], *p* = 0.114), with females demonstrating numerically higher magnification scores than males. All other sociodemographic variables, including age, year of study, educational level, residence, having children, and marital status, were non-significant predictors (all *p* > 0.05). (Supplementary Table S4)

The Helplessness subscale regression model identified gender and residence as significant predictors. Female participants reported significantly higher helplessness scores compared to males (B = 1.973, 95% CI [0.90, 2.96], *p* < 0.001), representing the strongest predictor in the model. Among residence categories, participants from medium-sized settlements (5000–50,000 people) showed significantly higher helplessness scores relative to rural residents (B = 1.865, 95% CI [0.50, 3.19], *p* = 0.006). Small settlement residents reported higher helplessness (B = 0.747, 95% CI [−0.59, 2.06], *p* = 0.263), while urban residents showed no significant difference from rural participants. All other variables, including age, year of study, educational level, employment status, income, having children, and marital status, were non-significant predictors (all *p* > 0.05). (Supplementary Table S5)

For the Rumination subscale, both gender and employment status emerged as significant predictors. Female participants exhibited significantly higher rumination scores than males (B = 1.269, 95% CI [0.04, 2.54], *p* = 0.033). Employed individuals demonstrated significantly elevated rumination scores compared to student/unemployed participants (B = 2.549, 95% CI [0.37, 4.42], *p* = 0.014), representing the strongest predictor in this model. Small settlement residence was associated with higher rumination scores, but without reaching statistical significance (B = 1.227, 95% CI [−0.03, 2.50], *p* = 0.060). All remaining sociodemographic variables, including age, year of study, educational level, other residence categories, income, having children, and marital status, were non-significant predictors (all *p* > 0.05). (Supplementary Table S6)

The overall TCS Total score regression analysis revealed gender as the sole significant predictor among all sociodemographic variables examined. Female participants demonstrated significantly higher total terrorism catastrophizing scores compared to males (B = 3.986, 95% CI [1.649, 6.529], *p* < 0.001). Several variables showed higher scores in small settlement residence (B = 2.443, 95% CI [−0.339, 5.580], *p* = 0.069), medium-sized settlement residence (B = 2.580, 95% CI [−0.391, 6.210], *p* = 0.092), and employment status (B = 3.240, 95% CI [−0.424, 6.855], *p* = 0.077). All other predictors, including age, year of study, educational level, urban residence, income categories, having children, and marital status variables, were non-significant (all *p* > 0.05). The consistent pattern across all TCS measures indicates that gender represents the most robust demographic predictor of terrorism catastrophizing in this sample. (Supplementary Table S7)

Bootstrapped regression analysis for the Behavioral subscale identified employment status as the only significant predictor among sociodemographic variables. Employed participants demonstrated significantly higher behavioral change scores compared to student/unemployed individuals (B = 1.929, 95% CI [0.202, 3.564], *p* = 0.025). Urban residence was associated with lower behavioral change scores (B = −0.962, 95% CI [−2.076, 0.018], *p* = 0.097). Having children also approached significance (B = −1.504, 95% CI [−3.435, 0.011], *p* = 0.100), indicating that parents could potentially exhibit fewer behavioral changes than those without children, although this conclusion would require further exploration. All other sociodemographic variables, including age, year of study, gender, educational level, other residence categories, income levels, and marital status variables, were non-significant predictors (all *p* > 0.05). (Supplementary Table S8)

For the Habitual subscale, urban residence emerged as the sole significant predictor. Participants residing in urban areas (>50,000 people) reported significantly lower habitual change scores compared to rural residents (B = −1.50, 95% CI [−2.57, −0.52], *p* = 0.011). Employment status was not statistically significant (B = 1.24, 95% CI [−0.18, 2.62], *p* = 0.070), though the direction of the effect suggested that employed individuals may potentially be more likely to modify their habitual patterns due to terrorism concerns. All remaining variables, including age, year of study, gender, educational level, other residence categories, income levels, having children, and marital status, were non-significant predictors (all *p* > 0.05). (Supplementary Table S9)

The Total Behavioral scale regression model revealed two significant predictors: employment status and urban residence. Employed participants exhibited significantly higher total behavioral change scores than student/unemployed individuals (B = 3.18, 95% CI [0.11, 6.02], *p* = 0.028). Conversely, urban residents demonstrated significantly lower behavioral modification scores compared to rural residents (B = −2.46, 95% CI [−4.26, −0.79], *p* = 0.010). Being in a romantic relationship was not statistically significant (B = −1.79, 95% CI [−4.00, 0.60], *p* = 0.148), though the direction of the effect suggested that individuals in a relationship may possibly engage in fewer terrorism-related behavioral changes. All other sociodemographic variables, including age, year of study, gender, educational level, other residence categories, income levels, having children, and other marital status categories, were non-significant predictors (all *p* > 0.05). These findings suggest that employment status and residential context are key factors modifying terrorism-related behavioral adaptations, with employed individuals showing greater behavioral sensitivity to terrorism concerns while urban residents demonstrate less behavioral modification overall. (Supplementary Table S10)

## 4. Discussion

This study examined terrorism catastrophizing in a cohort of Croatian nursing students—a group seldom studied in relation to terrorism-related cognitive processes. The mean TCS score of 38.4 (SD 8.0) reflected a moderate degree of catastrophizing. These findings are comparable to those observed in population samples outside active conflict zones [7,8]. Across the three cognitive subscales, helplessness emerged as the most prominent dimension, followed by rumination and magnification. A similar profile has been reported in higher-risk countries, where greater perceived threat was associated with greater distress, although in some cases, it coexisted with signs of adaptation or resilience [9,10,23]. While heightened helplessness is often associated with maladaptive coping, in some contexts it may represent a functional cognitive distancing strategy—an acknowledgment that the threat cannot be mitigated through individual action, which in turn can reduce unnecessary personal strain. This interpretation, however, would need further study.

In the Croatian context, where the risk is currently low but the memory of armed conflict remains, the prominence of helplessness may indicate that historical trauma—even when decades old—may possibly sustain vulnerability to perceived uncontrollable dangers [8].

Gender-related differences were also noticeable in our study, with female students scoring higher on all TCS dimensions. This finding is consistent with prior research showing that women tend to report greater fear of terrorism and higher perceived risk than men [24]. For example, a 2009 survey in the U.S. found that women, in addition to being more afraid of terrorism, also engaged in more information-seeking and avoidance behaviors [24]. Beyond the specific domain of terrorism, this is also congruent with similar studies [25]. Explanations for this disparity often reference differences in threat perception, coping strategies, and culturally shaped social roles. Within nursing education, evidence on gender and stress is mixed. For instance, Senturk and Dogan observed greater stress in female nursing students in Turkey [14], while others report no significant gender differences [26,27]. This suggests that gender-targeted approaches might be warranted in addressing terrorism fears. In line with methodological recommendations, such differences should be interpreted with regard to both their statistical significance and their practical relevance, while further investigation is needed to understand any protective factors in male students.

Employment status also proved significant. Students who were employed alongside their study reported higher scores on magnification and rumination subscales, as well as more frequent behavioral changes in response to terrorism threat. One possible interpretation is that employment—especially in healthcare or public-facing positions—may increase exposure to conversations and experiences related to societal risk, which could reinforce threat-related cognitive schemas. Another possibility is that the added workload and chronic stress associated with such employment [28,29] may deplete psychological resources, thereby increasing general vulnerability to unhelpful and possibly maladaptive cognitive responses [15]. While our data cannot pinpoint the exact mechanism, the association between employment and elevated catastrophizing suggests that extra support may be needed for nursing students who are employed during their studies.

An unexpected finding involved the place of residence. Urban students reported fewer habitual and overall behavioral changes in response to the terrorism threat, a contrast to patterns in some European studies, where residents of major cities often consider themselves more likely targets and thus might be expected to show greater vigilance or worry [18]. In the Croatian context, one possible explanation is that living in a city may normalize a certain level of security alertness, due to routine safety measures and public awareness campaigns, which could paradoxically lead urban students to feel more secure, thus not altering their behavior as much. This remains a tentative hypothesis requiring further empirical examination. By contrast, students in smaller communities—who may feel more removed from any possible terror event—might perceive any terrorism news as a more extraordinary threat to their local stability, which could prompt disproportionate adjustments (e.g., avoiding travel or public gatherings even if the risk is remote). It is also possible that urban students may have greater trust in institutional protection (police, security services) given the visible presence of such infrastructure in cities, although these explanations remain speculative and warrant further investigation.

We also found that income level was inversely related to one aspect of catastrophizing: students from higher-income households scored significantly lower on the magnification subscale. In practical terms, those with more financial security were less likely to exaggerate the likelihood or impact of a potential terrorism event. This aligns with the idea that economic and personal resources can buffer individuals against threat perceptions [15]. Those with greater economic resources may feel better equipped to manage potential consequences, which could reduce the intensity of catastrophic thoughts.

Importantly, we observed that terrorism catastrophizing was moderately correlated with self-reported behavioral changes. Students who scored higher on the TCS were more likely to have changed their daily habits (e.g., avoiding certain activities or places) due to terrorism concerns. This underscores the functional relevance of catastrophic cognitions: it is not merely an abstract mindset, but one that can translate into concrete avoidance behaviors. This finding is also consistent with cognitive–behavioral models of anxiety disorders, which posit that exaggerated threat appraisals prompt avoidance behaviors and hypervigilance [30].

In our context, a nursing student with high catastrophizing might, for example, avoid attending large public events or feel anxious about using public transport—behaviors that could have downstream effects on their social life and even clinical training (if they avoid certain hospital rotations or community health assignments perceived as risky). Given that our participants are future healthcare providers, sustained patterns of avoidance or anxiety could potentially impact their professional readiness—for instance, willingness to work in emergency departments or respond to mass casualty incidents could be compromised if terrorism-related anxiety is high. This highlights a need to address catastrophic thinking not only for students’ personal well-being but also for the robustness of the healthcare workforce in crisis situations.

From a nursing education perspective, our findings have several implications. One is that it may be beneficial to integrate training into nursing curricula that fosters adaptive threat appraisal and coping skills among nursing students. Ensuring that future nurses maintain a balanced view of risks is critical, given their role in emergency response and public health messaging. Educational curricula could incorporate evidence-based stress management techniques and resilience-building interventions to help students handle anxiety about low-probability, high-impact events like terrorism. Additionally, introducing media literacy modules may empower students to critically evaluate news reports and avoid sensationalism-induced panic [31].

On a broader level, the results underscore the importance of responsible risk communication in the public sphere. Excessive media exposure and alarmist reporting on terrorism have been linked to heightened anxiety in populations [32]. Public health and government communication strategies should therefore aim to reduce sensationalism without downplaying real risks. Providing clear, factual, and reassuring information can lower the likelihood of disproportionate fear responses across the student population and the public at large. Studies during the COVID-19 pandemic showed that constant consumption of upsetting news contributed to mental distress, suggesting a similar dynamic may occur with terrorism news [33]. Thus, balanced messaging—for example, highlighting successful counter-terrorism measures or the low absolute risk in a given area—could mitigate unwarranted anxiety.

Taken together, these findings point to actionable steps: incorporating terrorism-related stress management and critical media appraisal content into nursing education and framing public messages to avoid inadvertent fear escalation. Such efforts acknowledge that psychological resilience against terror threats is not just an individual trait, but something that can be strengthened through education and prudent communication. Given evidence that continuous alarmist media can amplify public anxiety [32] even creating a self-perpetuating “cycle of distress” after collective trauma [34]—building cognitive resilience is as important as improving physical security.

Future research could address several priorities. Comparative studies between post-conflict and high-risk regions may help clarify the influence of historical trauma on terrorism catastrophizing. Longitudinal designs would be valuable for determining whether elevated catastrophizing predicts occupational outcomes in nursing graduates. Experimental trials could test the efficacy of cognitive–behavioral or psychoeducational interventions in reducing unhelpful cognitive patterns and related behaviors. Finally, examining how terrorism catastrophizing interacts with other forms of threat appraisal—such as pandemic-related fear—could inform integrated preparedness training for healthcare students in an era of multiple, overlapping global risks.

### Study Strengths and Limitations

This study is, to our knowledge, the first to examine terrorism catastrophizing among nursing students in Croatia, addressing a notable gap in the literature on terrorism-related cognitive processes in post-conflict, low-risk European contexts. Methodologically, the application of bootstrapped regression analyses strengthened statistical inferences by mitigating the impact of potential non-normality in the data.

However, several limitations should be acknowledged. While the Croatian version of the TCS demonstrated good internal consistency in this study, its construct validity was examined in a separate study on a larger sample, which is being published independently. The cross-sectional design precludes any inference about causality between sociodemographic factors and terrorism catastrophizing, and longitudinal data would be needed to establish temporal relationships. Although the sample was sizeable, it was limited to nursing students from a single country, restricting generalizability to other healthcare disciplines or cultural contexts. Effect sizes were not calculated for group comparisons, and the interpretation of differences is therefore based on statistical significance rather than magnitude. The mixed-mode survey approach and the use of snowball sampling may have introduced biases, such as differences in response style, social desirability effects, or overrepresentation of more socially connected students. Finally, as the study was conducted in a low-risk, post-conflict setting, findings may not extrapolate to high-risk regions or populations with direct exposure to terrorism.

## 5. Conclusions

Terrorism catastrophizing among Croatian nursing students was moderate, influenced by gender, employment, and residential context. These findings underline that even in low-risk countries, nursing students may carry significant psychological concerns related to global security threats. For nursing education and public health, this emphasizes the value of targeted mental health support and tailored risk communication strategies to strengthen student resilience and professional readiness. Given the pivotal role of nurses in future healthcare responses, enhancing their ability to cope with such fears is of societal importance. Future research should build on these results with longitudinal and cross-national studies to clarify causal pathways and inform interventions that foster resilience in the healthcare workforce.

## Figures and Tables

**Table 1:** Participant Sociodemographic Characteristics.

Variable	n or Mean (SD), Range	% or Median [IQR]
**Age (years)**	24.0 (6.0), 18–53	22 [21–24]
**Year of Study**		
1st year	84	24.10%
2nd year	108	31.00%
3rd year	131	37.60%
4th year	12	3.40%
5th year	13	3.70%
**Gender**		
Male	49	14.10%
Female	299	85.90%
**Last Completed Educational Level**		
Secondary school	321	92.20%
College or university	27	7.80%
**Place of Residence**		
Rural area	73	21.00%
Small settlement (<5000 people)	53	15.20%
Medium-size settlement (5 k–50 k)	70	20.10%
Urban (>50,000 people)	152	43.70%
**Employment Status**		
Student/unemployed	188	54.00%
Employed	160	46.00%
**Average Monthly Income**		
<677 euros	150	43.10%
677–1000 euros	48	13.80%
1001–2000 euros	126	36.20%
>2000 euros	24	6.90%
**Do You Have Any Children?**		
No	305	87.60%
Yes	43	12.40%
**Marital Status**		
Single	205	58.90%
Cohabiting with a partner	73	21.00%
In a relationship	20	5.70%
Legally married	40	11.50%
Divorced/Widow	10	2.90%

**Table 2:** Behavioral and Habitual Changes due to Threat of Terrorism.

Item	Mean (SD), Range	Median [IQR]
Flying on commercial airplanes	1.9 (1.4), 1–5	1.0 [1.0–3.0]
Using public transportation	1.5 (1.0), 1–5	1.0 [1.0–1.0]
Going to public places	1.4 (0.9), 1–5	1.0 [1.0–1.0]
Voting in elections	1.4 (1.0), 1–5	1.0 [1.0–1.0]
Interacting with individuals of Middle Eastern or Arab descent	2.2 (1.5), 1–5	1.0 [1.0–3.0]
**Behavioral subscale**	8.3 (4.0), 5–25	7.0 [5.0–10.0]
Vacationing preferences	1.9 (1.2), 1–5	1.0 [1.0–3.0]
Working or attending school	1.6 (1.0), 1–5	1.0 [1.0–2.0]
Residential decisions	1.9 (1.3), 1–5	1.0 [1.0–3.0]
News consumption habits	2.0 (1.3), 1–5	1.0 [1.0–3.0]
**Habitual subscale**	7.5 (3.7), 4–20	6.0 [4.0–10.0]
**Total behavioral scale**	15.7 (6.7), 9–45	14.0 [10.0–19.0]

**Table 3:** Internal Consistency Reliability (Cronbach’s Alpha) for Study Scales.

Scale	Cronbach’s α	Number of Items
Magnification	0.820	3
Helplessness	0.715	5
Rumination	0.777	5
TCS (Total)	0.827	13
Behavioral	0.718	5
Habitual	0.786	4
Total Behavioral	0.810	9

**Table 4:** Group Differences in TCS Total and Behavioral Scale Scores by Sociodemographic Variables.

Category	TCS Total Mean ± SD	*p*-Value	Behavioral Scale Mean ± SD	*p*-Value
**Year of Study**				
1st year	39.2 ± 7.7	0.432	16.2 ± 6.8	0.557
2nd year	38.3 ± 9.2		15.7 ± 6.8	
3rd year	37.6 ± 7.2		15.7 ± 6.8	
4th year	39.3 ± 7.8		16.0 ± 4.9	
5th year	40.5 ± 7.3		12.5 ± 3.1	
**Gender**				
Male	35.0 ± 7.9	**0.001**	16.4 ± 8.2	0.856
Female	38.9 ± 7.9		15.6 ± 6.4	
**Last Completed Educational Level**				
Secondary school	38.3 ± 8.1	0.199	15.8 ± 6.8	0.893
College or university	39.7 ± 7.1		14.7 ± 4.6	
**Place of Residence**				
Rural area	37.7 ± 9.1	0.075	16.2 ± 6.7	**0.01**
Small settlement (<5000)	40.1 ± 6.1		17.4 ± 8.0	
Medium (5000–50,000)	40.1 ± 8.0		17.1 ± 6.9	
Urban (>50,000)	37.2 ± 7.9		14.5 ± 5.8	
**Employment Status**				
Student/unemployed	38.0 ± 8.2	0.445	14.4 ± 5.9	**<0.001**
Employed	38.8 ± 7.8		17.2 ± 7.1	
**Average Monthly Income**				
<677 euros	38.4 ± 8.1		14.4 ± 5.6	
677–1000 euros	38.5 ± 8.6		15.7 ± 7.1	
1001–2000 euros	38.8 ± 7.9		17.4 ± 7.3	
>2000 euros	36.0 ± 6.7	0.339	15.5 ± 6.1	**0.01**
**Children**				
No	38.4 ± 7.9	0.8	15.9 ± 6.8	0.611
Yes	37.8 ± 8.7		14.9 ± 5.6	
**Marital Status**				
Single	38.3 ± 8.1	0.609	15.6 ± 6.7	
Cohabiting with a partner	38.7 ± 7.8		17.3 ± 7.1	
In a relationship	39.7 ± 7.2		13.4 ± 4.4	
Legally married	36.8 ± 8.1		14.5 ± 5.7	
Divorced/Widow	40.3 ± 9.1		17.3 ± 7.9	0.159

**Table 5:** Spearman’s Correlations Between Age, TCS Total, and Behavioral Scale Scores.

Variable	1. Age	2. TCS Total	3. Behavioral Scale
1. Age	—		
2. TCS Total	−0.06 (0.309)	—	
3. Behavioral Scale	0.06 (0.275)	0.27 (<0.001) **	—

Note. Values are Spearman’s rho correlation coefficients with *p*-values in parentheses. n = 333–348, depending on variable pair. ** Correlation is significant at the 0.01 level (2-tailed).

## Data Availability

The original contributions presented in this study are included in the article/Supplementary Materials. Further inquiries can be directed to the corresponding author.

## Supplementary Materials

The following supporting information can be downloaded at: https://www.mdpi.com/article/10.3390/healthcare13182323/s1, Table S1: STROBE Statement—Checklist of items that should be included in reports of cross-sectional studies; Table S2: Descriptive Statistics for Terrorism Catastrophizing Scale (TCS) Items and Subscales; Table S3: Tests of normality; Table S4: Bootstrapped Regression Coefficients Predicting Magnification; Table S5: Bootstrapped Regression Coefficients Predicting Helplessness; Table S6: Bootstrapped Regression Coefficients Predicting Rumination; Table S7. Bootstrap Coefficients Table for TCS total; Table S8: Bootstrapped Regression Coefficients Predicting Behavioral Scale; Table S9: Bootstrap Estimates of Regression Coefficients for habitual subscale; Table S10: Bootstrap Estimates of Regression Coefficients for total behavioral scale.

## Abbreviations

The following abbreviations are used in this manuscript:

TCS	Terrorism Catastrophizing Scale
BCa	Bias-Corrected and Accelerated
SD	Standard Deviation
Mdn	Median
IQR	Interquartile Range
α	Cronbach’s Alpha
CI	Confidence Interval
df	Degrees of Freedom
rs	Spearman’s rank-order correlation coefficient
*p*	*p*-value (statistical significance level)
STROBE	Strengthening the Reporting of Observational Studies in Epidemiology
