# Supplementary material for: Terrorism Catastrophizing and Sociodemographic Correlates Among Croatian Nursing Students: A Cross-Sectional Study
Source: Healthcare (Basel). 2025 Sep 16;13(18):2323. doi: 10.3390/healthcare13182323 (PMC12469285; doi:10.3390/healthcare13182323)
Supplement: Supplementary file 1 [file healthcare-13-02323-s001.zip › healthcare-3845517-supplementary.pdf]

Supplementary Materials

Table S1. STROBE Statement — Checklist of items that should be included in reports of cross-sectional studies

Table S2. Descriptive Statistics for Terrorism Catastrophizing Scale (TCS) Items and Subscales

Table S3. Tests of normality

Table S4. Bootstrapped Regression Coefficients Predicting Magnification

Table S5. Bootstrapped Regression Coefficients Predicting Helplessness

Table S6. Bootstrapped Regression Coefficients Predicting Rumination

Table S7. Bootstrap Coefficients Table for TCS total

Table S8. Bootstrapped Regression Coefficients Predicting Behavioral Scale

Table S9. Bootstrap Estimates of Regression Coefficients for habitual subscale

Table S10. Bootstrap Estimates of Regression Coefficients for total behavioral scale

Table S1. STROBE Statement — Checklist of items that should be included in reports of cross-sectional studies

	Item No	Recommendation	Location in Manuscript
**Title and abstract**	1	(*a*) Indicate the study's design with a commonly used term in the title or the abstract	Title page, Abstract (p. 1)
		(*b*) Provide in the abstract an informative and balanced summary of what was done and what was found	Abstract (p. 1)
		**Introduction**	
Background/rationale	2	Explain the scientific background and rationale for the investigation being reported	Introduction, first paragraph (p. 1)
Objectives	3	State specific objectives, including any prespecified hypotheses	Introduction, last paragraph (p. 2)
		**Methods**	
Study design	4	Present key elements of study design early in the paper	Methods 2.1. Study design (p. 2)
Setting	5	Describe the setting, locations, and relevant dates, including periods of recruitment, exposure, follow-up, and data collection	Methods 2.3. Data collection (p. 3)
Participants	6	(*a*) Give the eligibility criteria, and the sources and methods of selection of participants	Methods 2.2. Participants (p. 3)
Variables	7	Clearly define all outcomes, exposures, predictors, potential confounders, and effect modifiers. Give diagnostic criteria, if applicable	Methods 2.4. Instruments (p. 3-4)
Data sources/ measurement	8*	For each variable of interest, give sources of data and details of methods of assessment (measurement). Describe comparability of assessment methods if there is more than one group	Methods 2.4. Instruments (p. 3-4)
Bias	9	Describe any efforts to address potential sources of bias	Discussion 4.1. Limitations (p. 13-14)
Study size	10	Explain how the study size was arrived at	Methods 2.2. Participants (p. 3)
Quantitative variables	11	Explain how quantitative variables were handled in the analyses. If applicable, describe which groupings were chosen and why	Methods 2.4. Instruments (p. 3-4) 2.5. Data Analysis (p. 4)
Statistical methods	12	(*a*) Describe all statistical methods, including those used to control for confounding	Methods 2.5. Data Analysis (p. 4)
		(*b*) Describe any methods used to examine subgroups and interactions	Methods 2.5. Data Analysis (p. 4)
		(*c*) Explain how missing data were addressed	Methods 2.2. Participants (p. 3)
		(*d*) If applicable, describe analytical methods taking account of sampling strategy	Methods 2.2. Participants (p.3)
		(*e*) Describe any sensitivity analyses	
		**Results**	
Participants	13*	(a) Report numbers of individuals at each stage of study—eg numbers potentially eligible, examined for eligibility, confirmed eligible, included in the study, completing follow-up, and analysed	Methods 2.2. Participants (p. 3)
		(b) Give reasons for non-participation at each stage	Not applicable (voluntary anonymous survey)

		(c) Consider use of a flow diagram	
Descriptive data	14*	(a) Give characteristics of study participants (eg demographic, clinical, social) and information on exposures and potential confounders	Results (p. 5)<br>Table 1.
		(b) Indicate number of participants with missing data for each variable of interest	Results (p. 5)
Outcome data	15*	Report numbers of outcome events or summary measures	Results (p. 5-11)
Main results	16	(*a*) Give unadjusted estimates and, if applicable, confounder-adjusted estimates and their precision (eg, 95% confidence interval). Make clear which confounders were adjusted for and why they were included	Results (p. 5-11)
		(*b*) Report category boundaries when continuous variables were categorized	Methods<br>2.2. Participants (p. 3)
		(*c*) If relevant, consider translating estimates of relative risk into absolute risk for a meaningful time period	Not applicable
Other analyses	17	Report other analyses done—eg analyses of subgroups and interactions, and sensitivity analyses	Results (p. 5-11)
		**Discussion**	
Key results	18	Summarise key results with reference to study objectives	Discussion,<br>first paragraph (p. 11)
Limitations	19	Discuss limitations of the study, taking into account sources of potential bias or imprecision. Discuss both direction and magnitude of any potential bias	Discussion<br>4.1. Limitations (p. 13-14)
Interpretation	20	Give a cautious overall interpretation of results considering objectives, limitations, multiplicity of analyses, results from similar studies, and other relevant evidence	Discussion (p. 11-13)
Generalisability	21	Discuss the generalisability (external validity) of the study results	Discussion<br>4.1. Limitations (p. 13)
		**Other information**	
Funding	22	Give the source of funding and the role of the funders for the present study and, if applicable, for the original study on which the present article is based	Funding statement (p. 14)

*Give information separately for exposed and unexposed groups.

Note: An Explanation and Elaboration article discusses each checklist item and gives methodological background and published examples of transparent reporting. The STROBE checklist is best used in conjunction with this article (freely available on the Web sites of PLoS Medicine at http://www.plosmedicine.org (accessed on 5 September 2025)/, Annals of Internal Medicine at http://www.annals.org (accessed on 5 September 2025)/, and Epidemiology at http://www.epidem.com/ (accessed on 5 September 2025)). Information on the STROBE Initiative is available at www.strobe-statement.org (accessed on 5 September 2025).

Table S2. Descriptive Statistics for Terrorism Catastrophizing Scale (TCS) Items and Subscales

Item / Subscale	Mean (SD)	Median [IQR]
Difficulty keeping the threat of terrorism out of mind	2.8 (1.2)	3.0 [2.0–4.0]
Little I can do to protect myself from terrorism	3.6 (1.1)	4.0 [3.0–4.0]
Frequently think about threat of future terrorism	2.7 (1.2)	3.0 [2.0–4.0]
Nothing I can do to defend against future attacks	3.3 (1.0)	3.0 [3.0–4.0]
Threat of terrorism does not enter my mind often	2.4 (1.1)	2.0 [2.0–3.0]
Worry terrorism will worsen with time	3.5 (1.1)	4.0 [3.0–4.0]
Feel helpless protecting myself	3.4 (1.0)	3.0 [3.0–4.0]
Worry threat of terrorism will never end	3.3 (1.1)	3.0 [3.0–4.0]
Often dwell on future terrorism	2.0 (1.0)	2.0 [1.0–2.0]
Believe future is dark due to terrorism	3.0 (1.1)	3.0 [2.0–4.0]
Feel powerful in keeping self-safe	3.5 (1.0)	3.0 [3.0–4.0]
Frequently preoccupied with terrorism	1.9 (1.0)	2.0 [1.0–2.0]
Lack control in defending against terrorism	3.0 (1.1)	3.0 [2.0–4.0]
Magnification	9.8 (2.8)	10.0 [8.0–12.0]
Helplessness	16.8 (3.6)	17.0 [15.0–19.0]
Rumination	11.7 (4.0)	12.0 [9.0–14.0]
**TCS Total**	**38.4 (8.0)**	**38.0 [34.0–44.0]**

Table S3. Tests of normality

	Kolmogorov-Smirnov[a]			Shapiro-Wilk		
	**Statistic**	**df**	**Sig.**	**Statistic**	**df**	**Sig.**
Magnification	0.121	348	<0.001	0.958	348	<0.001
Helplessness	0.069	348	<0.001	0.982	348	<0.001
Rumination	00.069	348	<0.001	0.974	348	<0.001
TCS Total	0.074	348	<0.001	0.990	348	0.015
Behavioral	0.206	333	<0.001	0.809	333	<0.001
Habitual	0.175	333	<0.001	0.852	333	<0.001
Total behavioral scale	0.171	333	<0.001	0.872	333	<0.001

a. Lilliefors Significance Correction

Table S4. Bootstrapped Regression Coefficients Predicting Magnification

Predictor Variable	B [95% CI]	Bias	p
**Intercept**	8.121 [5.34, 10.61]	–0.064	< 0.001
Year of study	–0.036 [–0.41, 0.36]	0.002	0.860
Age	–0.056 [–0.13, 0.02]	0.001	0.130
Gender (Female)	0.744 [–0.09, 1.71]	0.010	0.114
Education (College/University)	0.554 [–0.97, 2.02]	0.002	0.450
Residence (Small settlement <5000)	0.469 [–0.48, 1.48]	0.017	0.357
Residence (Medium-size 5000–50,000)	–0.097 [–1.06, 1.01]	0.019	0.849
Residence (Urban >50,000)	–0.449 [–1.34, 0.49]	–0.004	0.328
Employment (Employed)	1.235 [–0.09, 2.41]	0.003	0.033
Monthly income (€677–1000)	–0.487 [–1.45, 0.45]	0.014	0.345
Monthly income (€1001–2000)	–1.227 [–2.48, 0.10]	–0.008	0.061
Monthly income (>€2000)	–1.410 [–2.76, 0.12]	–0.015	0.049
Has children (Yes)	0.656 [–0.96, 2.26]	0.008	0.409
Marital status (Cohabiting with partner)	0.097 [–0.63, 0.85]	–0.003	0.816
Marital status (In a relationship)	0.342 [–1.01, 1.61]	–0.047	0.606
Marital status (Legally married)	–0.363 [–1.94, 1.14]	0.009	0.605
Marital status (Divorced/Widow)	0.945 [–1.24, 2.95]	–0.042	0.332

Table S5. Bootstrapped Regression Coefficients Predicting Helplessness

Predictor Variable	B [95% CI]	Bias	p
**Intercept**	11.621 [8.42, 15.10]	0.077	< 0.001
Year of study	0.010 [–0.42, 0.44]	–0.007	0.968
Age	0.036 [–0.06, 0.17]	0.006	0.551
Gender (Female)	1.973 [0.90, 2.96]	–0.038	< 0.001
Education (College/University)	0.760 [–1.02, 2.25]	–0.011	0.339
Residence (Small settlement <5000)	0.747 [–0.59, 2.06]	–0.015	0.263
Residence (Medium-size 5000–50,000)	1.865 [0.50, 3.19]	0.008	0.006
Residence (Urban >50,000)	–0.088 [–1.16, 0.95]	–0.022	0.874
Employment (Employed)	–0.543 [–2.03, 0.72]	–0.091	0.474
Monthly income (€677–1000)	–0.035 [–1.23, 1.29]	0.030	0.954
Monthly income (€1001–2000)	0.740 [–0.94, 2.76]	0.075	0.359
Monthly income (>€2000)	–0.321 [–2.24, 1.65]	0.051	0.734
Has children (Yes)	–0.664 [–2.47, 1.05]	–0.063	0.482
Marital status (Cohabiting with partner)	–0.181 [–1.27, 0.85]	0.007	0.737
Marital status (In a relationship)	1.191 [–0.28, 2.58]	–0.038	0.107
Marital status (Legally married)	–0.054 [–2.13, 2.01]	0.029	0.958
Marital status (Divorced/Widow)	0.140 [–3.02, 3.23]	–0.019	0.910

Table S6. Bootstrapped Regression Coefficients Predicting Rumination

Predictor Variable	B [95% CI]	Bias	p
**Intercept**	6.725 [2.66, 10.28]	–0.139	< 0.001
Year of study	–0.093 [–0.63, 0.35]	–0.005	0.725
Age	–0.045 [–0.18, 0.10]	0.004	0.460
Gender (Female)	1.269 [0.04, 2.54]	0.026	0.033
Education (College/University)	0.392 [–1.52, 2.49]	0.049	0.666
Residence (Small settlement <5000)	1.227 [–0.03, 2.50]	–0.001	0.060
Residence (Medium-size 5000–50,000)	0.811 [–0.62, 2.37]	0.054	0.236
Residence (Urban >50,000)	0.135 [–1.02, 1.37]	0.027	0.820
Employment (Employed)	2.549 [0.37, 4.42]	–0.047	0.014
Monthly income (€677–1000)	–0.219 [–1.58, 1.11]	–0.022	0.751
Monthly income (€1001–2000)	–1.270 [–3.20, 0.98]	0.037	0.224
Monthly income (>€2000)	–0.552 [–3.04, 2.00]	0.031	0.649
Has children (Yes)	0.405 [–1.62, 2.29]	–0.040	0.707
Marital status (Cohabiting with partner)	0.004 [–1.04, 0.98]	–0.021	0.995
Marital status (In a relationship)	–0.245 [–1.85, 1.30]	–0.004	0.770
Marital status (Legally married)	–1.145 [–2.81, 0.55]	–0.033	0.204
Marital status (Divorced/Widow)	0.497 [–2.90, 3.53]	–0.065	0.773

Table S7. Bootstrap Coefficients Table for TCS total

Predictor	B (95% CI)	Bias	*p*-value
**Intercept**	26.467 (18.938, 33.278)	-0.235	< 0.001
Year of study	-0.118 (-1.084, 0.780)	-0.013	0.807
Age	-0.066 (-0.298, 0.211)	0.004	0.585
Gender (Female)	3.986 (1.649, 6.529)	0.018	< 0.001
Education (College/University)	1.706 (-3.548, 6.288)	0.131	0.413
Residence (Small settlement <5000)	2.443 (-0.339, 5.580)	0.098	0.069
Residence (Medium-size 5000–50,000)	2.580 (-0.391, 6.210)	0.182	0.092
Residence (Urban >50,000)	-0.402 (-2.941, 2.154)	0.045	0.776
Employment (Employed)	3.240 (-0.424, 6.855)	-0.068	0.077
Monthly income (€677–1000)	-0.742 (-3.750, 2.219)	0.061	0.618
Monthly income (€1001–2000)	-1.757 (-5.651, 2.200)	0.035	0.375
Monthly income (>€2000)	-2.284 (-5.937, 1.629)	-0.048	0.258
Has children (Yes)	0.398 (-4.298, 5.198)	0.074	0.842
Marital status (Cohabiting with partner)	-0.080 (-2.358, 2.076)	0.010	0.944
Marital status (In a relationship)	1.288 (-2.359, 4.599)	-0.016	0.450
Marital status (Legally married)	-1.562 (-6.183, 2.193)	-0.011	0.415
Marital status (Divorced/Widow)	1.582 (-4.709, 7.938)	-0.118	0.627

Table S8. Bootstrapped Regression Coefficients Predicting Behavioral Scale

Predictor	B (95% CI)	Bias	*p*-value
(Constant)	7.434 (3.771, 10.780)	-0.060	< .001
Year of study	0.126 (–0.380, 0.585)	–0.004	.611
Age	–0.061 (–0.199, 0.084)	0.007	.386
Gender (Female)	0.170 (–1.227, 1.426)	–0.000	.787
Education (College/University)	–1.100 (–3.065, 0.783)	–0.032	.242
Residence (Small settlement <5000)	0.799 (–0.627, 2.253)	–0.023	.304
Residence (Medium-size 5000–50,000)	0.256 (–1.164, 1.662)	0.005	.709
Residence (Urban >50,000)	–0.962 (–2.076, 0.018)	–0.025	.097
Employment (Employed)	1.929 (0.202, 3.564)	–0.029	.025
Monthly income (€677–1000)	0.389 (–0.946, 1.719)	–0.010	.575
Monthly income (€1001–2000)	0.788 (–1.010, 2.702)	0.001	.399
Monthly income (>€2000)	–0.129 (–2.135, 2.161)	0.005	.913
Has children (Yes)	–1.504 (–3.435, 0.011)	–0.115	.100
Marital status (Cohabiting with partner)	0.731 (–0.467, 1.990)	0.008	.253
Marital status (In a relationship)	–0.936 (–2.406, 0.530)	–0.027	.263
Marital status (Legally married)	0.287 (–1.317, 2.073)	0.012	.755
Marital status (Divorced/Widow)	2.061 (–1.929, 6.164)	0.027	.281

Table S9. Bootstrap Estimates of Regression Coefficients for habitual subscale

Predictor	B (95% CI)	Bias	p-value
(Constant)	7.40 (4.24, 11.18)	0.053	< 0.001
Year of study	-0.22 (-0.66, 0.22)	-0.007	0.343
Age	0.01 (-0.08, 0.11)	0.000	0.810
Gender (Female)	-0.48 (-1.87, 0.71)	0.006	0.445
Education (College/University)	-0.11 (-1.87, 1.62)	-0.023	0.899
Residence (Small settlement <5000)	0.12 (-1.24, 1.49)	-0.055	0.862
Residence (Medium-size 5000–50,000)	-0.15 (-1.38, 1.10)	-0.049	0.842
Residence (Urban >50,000)	-1.50 (-2.57, -0.52)	-0.035	0.011
Employment (Employed)	1.24 (-0.18, 2.62)	0.010	0.070
Monthly income (€677–1000)	0.86 (-0.50, 2.32)	0.022	0.227
Monthly income (€1001–2000)	0.52 (-0.81, 1.91)	-0.019	0.513
Monthly income (>€2000)	0.26 (-1.37, 2.07)	-0.023	0.782
Has children (Yes)	0.16 (-1.89, 2.37)	0.109	0.858
Marital status (Cohabiting with partner)	0.09 (-0.97, 1.21)	-0.009	0.877
Marital status (In a relationship)	-0.87 (-2.24, 0.36)	-0.011	0.188
Marital status (Legally married)	-1.37 (-2.99, 0.15)	-0.062	0.118
Marital status (Divorced/Widow)	0.21 (-3.43, 3.43)	-0.118	0.895

Table S10. Bootstrap Estimates of Regression Coefficients for total behavioral scale

Predictor	B (95% CI)	Bias	*p*-value
**Constant**	14.77 (9.46, 20.08)	-0.043	< 0.001
Year of study	-0.08 (-0.88, 0.73)	0.011	0.869
Age	-0.05 (-0.23, 0.16)	0.004	0.589
Gender	-0.29 (-2.57, 2.01)	0.038	0.786
Last completed educational level	-1.25 (-4.21, 1.40)	-0.082	0.402
Small settlement (<5000 people)	0.92 (-2.01, 3.65)	0.007	0.522
Medium-size settlement (5000–50,000 people)	0.17 (-1.84, 2.46)	0.017	0.874
Urban (city >50,000 people)	-2.46 (-4.26, -0.79)	-0.058	0.010
Employment status	3.18 (0.11, 6.02)	~0	0.028
Income = 677–1000 euros	1.25 (-1.12, 3.61)	-0.040	0.305
Income = 1001–2000 euros	1.34 (-1.24, 4.15)	-0.030	0.357
Income >2000 euros	0.14 (-3.13, 3.57)	-0.024	0.936
Do you have any children?	-1.38 (-5.01, 1.73)	0.036	0.363
Cohabiting with partner	0.86 (-0.91, 2.71)	-0.015	0.336
In a relationship	-1.79 (-4.00, 0.60)	-0.001	0.148
Legally married	-1.08 (-4.19, 1.92)	0.003	0.490
Divorced/Widow	2.28 (-3.54, 7.95)	-0.019	0.406

Note: Bias and CIs are based on 1,000 bootstrap samples. CI = Confidence Interval.
Bias values shown as "~0" are extremely small (e.g., -2.839E-5).
